# Draft genome sequence of *Pantoea anthophila* PGPR-34, a chrysanthemum-associated Rhizobacterium with implications for plant–microbe interactions

**DOI:** 10.1128/mra.00346-25

**Published:** 2025-08-25

**Authors:** Tanim Jabid Hossain

**Affiliations:** 1 Department of Biochemistry and Molecular Biology, University of Chittagong 54493, Chattogram, Bangladesh; 2 Laboratory for Health, Omics and Pathway Exploration–HOPE Lab, Chattogram, Bangladesh; University of Strathclyde, Glasgow, United Kingdom

**Keywords:** *Pantoea anthophila*, genome sequence, plant–microbe interaction, rhizobacteria, nutrient mobilization, stress response

## Abstract

The genome sequence of *Pantoea anthophila* PGPR-34, isolated from chrysanthemum rhizosphere, comprises 4.78 Mb with 4,463 predicted protein-coding genes and 56.8% G + C content. Annotation identified genes predicted to mediate nutrient acquisition, abiotic stress tolerance, and plant–microbe interactions, providing a valuable genomic resource for exploring plant-beneficial mechanisms and advancing sustainable agriculture.

## ANNOUNCEMENT

Members of *Pantoea* are well-known for their ecological versatility and beneficial interactions with plants (1–3). *P. anthophila* PGPR-34 was isolated from the rhizosphere soil collected from several chrysanthemum plants in a private garden near the North campus of the University of Chittagong (coordinates: 22°28′49.4″N 91°47′39.7″E) (4). Root-attached soil was suspended in sterile water, serially diluted, and plated on Luria–Bertani (LB) and tryptone soya agar plates. Pure cultures were obtained via repeated streaking and maintained aerobically at 30°C. Taxonomic identification based on 16S rRNA gene sequence (GenBank OQ845745.1) demonstrated 99.78% identity with the type strain *P. anthophila* LMG 2558 (EF688010) (5). Given its *in vitro* plant-beneficial traits such as auxin production, phosphate solubilization, and ammonia production (5), genome sequencing was undertaken to elucidate underlying genetic determinants and inform future research on its ecological role and strain optimization.

Whole-genome sequencing of *P. anthophila* PGPR-34 was performed at Invent Technologies Ltd. (Bangladesh). A preculture was initiated by reviving the strain from a −80°C glycerol stock and used to inoculate fresh LB broth. After incubation at 30°C and 180 rpm, genomic DNA was extracted using the Wizard Genomic DNA Purification Kit (Promega). Sequencing was performed on the Illumina iSeq 100 platform (151-cycle paired-end kit) with on-instrument adapter trimming enabled, yielding 840,726 read pairs (mean length 114 bp; ~42× coverage). Reads were quality‐checked with FastQC  v0.12.1 (6) and trimmed using Trim Galore  v0.6.7 (7). *De novo* assembly was carried out with Unicycler v0.5.1 (8), excluding contigs <500 bp, and the assembly quality was evaluated using QUAST v5.3.0 (9). Unless specified otherwise, default parameters were applied in all applications. Assembly completeness, average nucleotide identity (ANI), and coding ratio (the percentage of the total genome length comprised of coding sequences) were determined using DFAST v.1.6.0 (24 March 2022) as previously described (10–12). The resulting draft genome of *P. anthophila* PGPR-34 comprised 43 contigs, with an N50 value of 267,216 bp and the largest contig measuring 526,702 bp. The genome size was 4,779,894 bp with a G + C content of 56.8%, matching other *P. anthophila* genomes in GenBank (4.41–4.77 Mb, ~57% G + C). The assembly showed 99.67% completeness and an 87.3% coding ratio. ANI analysis revealed 98.65% identity with the reference genome of the type strain *P. anthophila* LMG 2558 (GenBank GCA_006494375.1), confirming species-level identification.

Genome annotation using Prokka v1.14.6 (13) and Bakta v1.11.0 (14) predicted 4,463 protein-coding sequences, 3 rRNAs, 32 tRNAs, 1 tmRNA, and 40 ncRNAs. Functional assignment by RAST (RASTtk pipeline) (15) and BlastKOALA v3.1 (16) identified genes predicted to be involved in plant–microbe interactions, including nutrient mobilization (e.g., phosphate solubilization, nitrate and sulfate assimilation, and siderophore biosynthesis), secondary metabolism (e.g., biosynthesis of organic acids, γ-aminobutyric acid, and volatile compounds), and stress responses (oxidative, osmotic, periplasmic, and detoxification). Genes related to motility and chemotaxis, including those for flagellar biosynthesis, assembly, and regulatory proteins, were also identified. Overall, the genome sequence of *P. anthophila* PGPR-34 provides a valuable resource for further genetic and functional investigations and lays the groundwork for future studies on the ecological adaptability and niche specialization of plant-associated bacteria.

## Data Availability

This Whole Genome Shotgun project (PRJNA1246346) has been deposited in GenBank under accession number JBMUHZ000000000. The version described in this paper is JBMUHZ000000000.1 and consists of sequences JBMUHZ010000001-JBMUHZ010000043. Raw sequence reads are available in the NCBI Sequence Read Archive (SRA) under accession number SRR32973692. Annotation outputs are accessible at doi.org/10.6084/m9.figshare.29262614.
